# Supplementary material for: Individual characteristics, including olfactory efficiency, age, body mass index, smoking and the sex hormones status, and food preferences of women in Poland
Source: PeerJ. 2022 Jun 15;10:e13538. doi: 10.7717/peerj.13538 (PMC9206430; doi:10.7717/peerj.13538)
Supplement: Supplemental Information 1 [file peerj-10-13538-s001.pdf]

Potrawy z ryb

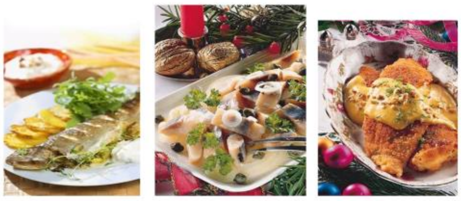

Pstrąg z rusztu | Śledziki | Ryba w panierce

1

Potrawy z jajek

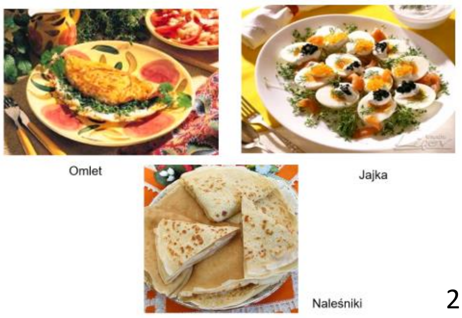

Omlet | Jajka | Naleśniki

2

Desery

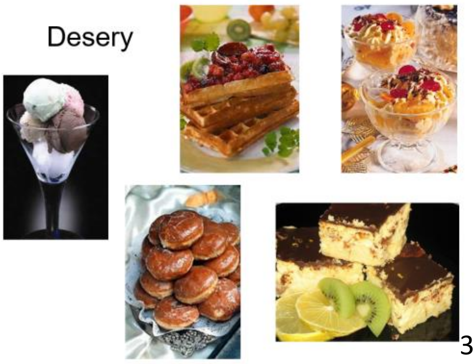

3

Wyroby czekoladowe

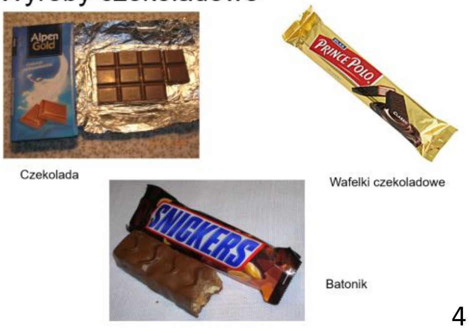

Czekolada | Wafelki czekoladowe | Batonik

4

Słodycze

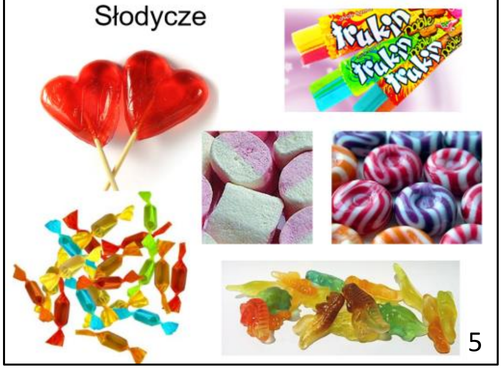

5

Chipsy, chrupki

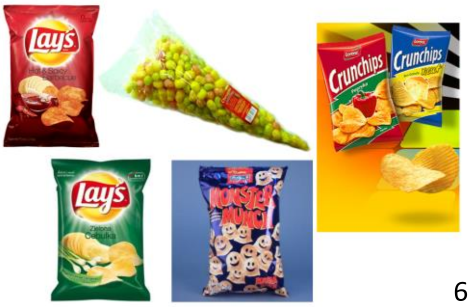

6

Potrawy mączne

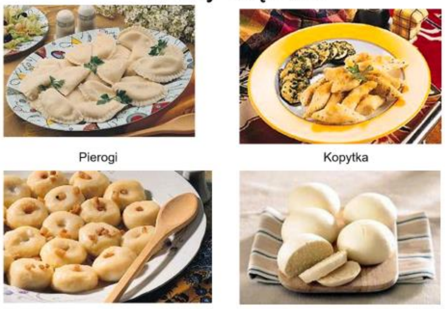

Pierogi | Kopytka | Kluski śląskie | Kluski na parze

7

Makarony

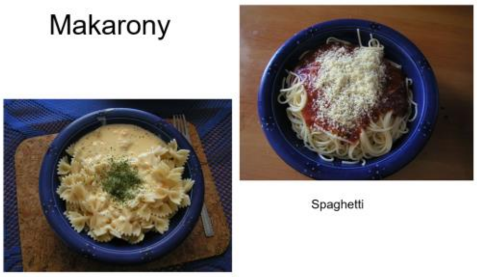

Spaghetti | Makaron z sosem

8

Zupy mleczne

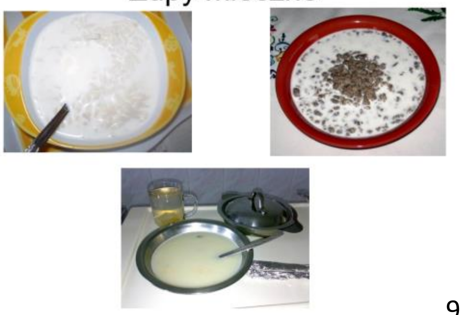

9

Produkty mleczne

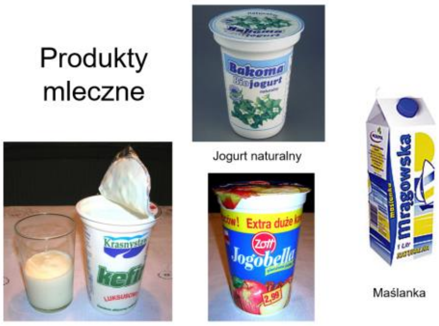

Jogurt naturalny | Kefir | Jogurt owocowy | Maślanka

10

Sery

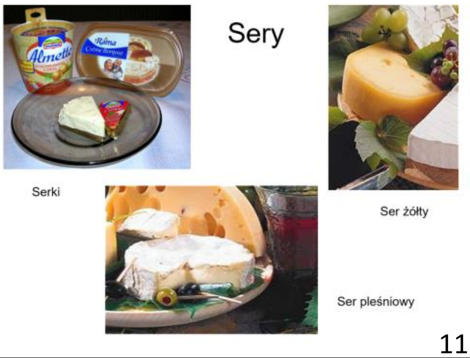

Serki | Ser żółty | Ser pleśniowy

11

Warzywa, surówki i sałatki

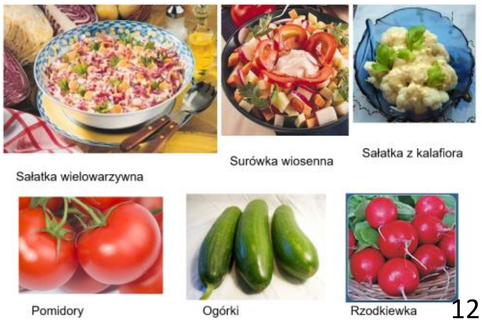

Sałatka wielowarzywna | Surówka wiosenna | Sałatka z kalafiora | Pomidory | Ogórki | Rzodkiewka

12

Owoce

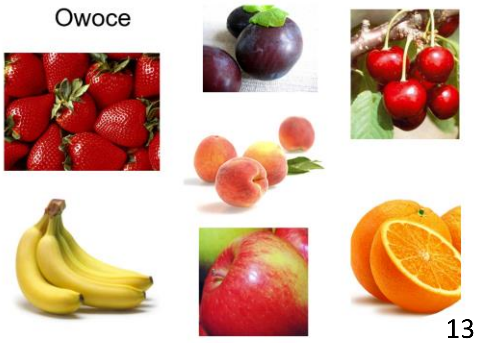

13

Wędliny

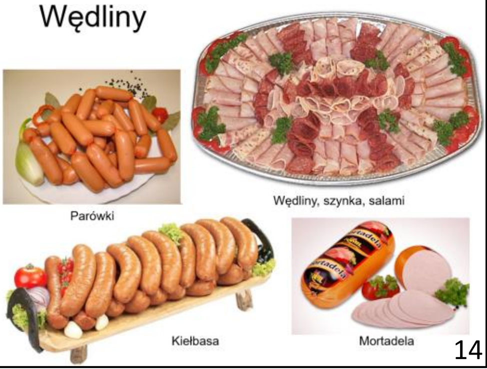

Parówki | Wędliny, szynka, salami | Kiełbasa | Mortadela

14

Mięso wołowe wieprzowe i cielęce

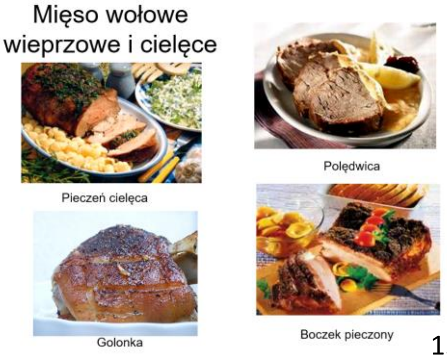

Pieczeń cielęca | Polędwica | Golonka | Boczek pieczony

15

Mięso drobiowe

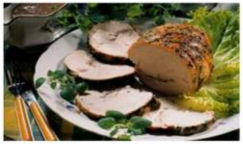
Pierś z indyka

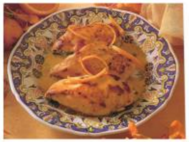
Pierś z kurczaka

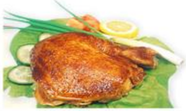
Udko z kurczaka

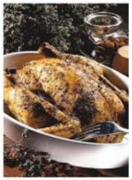
Pieczeń z indyka

16

Pieczywo

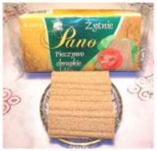

Pieczywo chrupkie

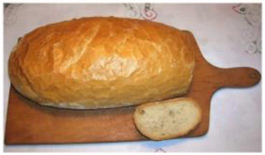
Chleb

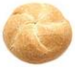
Kajzerka

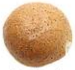
Grahamka

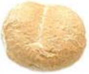
Bułka

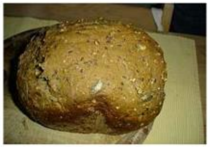
Chleb wiejski

17

Fast food

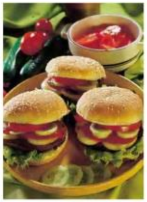
Hamburger

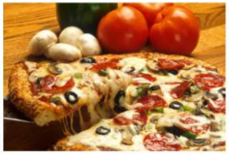
Pizza

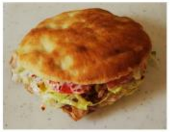
Kebab

18

Słone przekąski

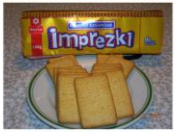

Krakersy

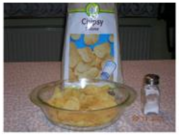
Chipsy

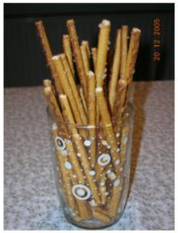
Paluszki

19

Kwaśne dodatki

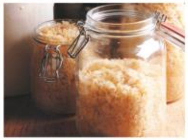
Kapusta kiszona

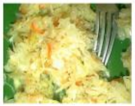

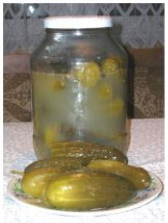
Ogórki kiszone

20

Rosół

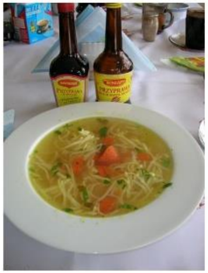

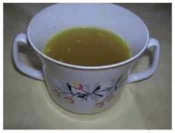

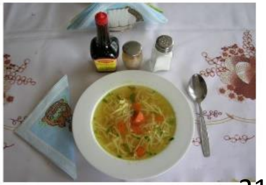

21

Zupy

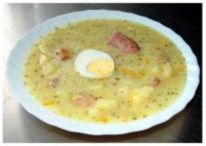
Żurek

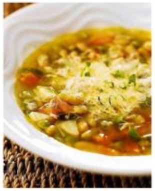
Zupa jarzynowa

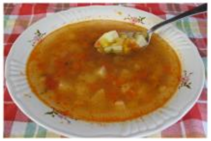
Zupa ziemniaczana

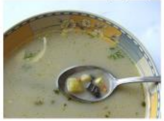
Zupa grzybowa

22

Ostre potrawy

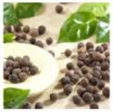
Pieprz

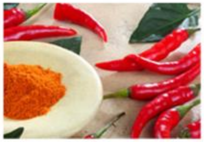
Ostre papryczki

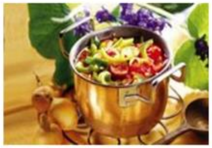
Leczo

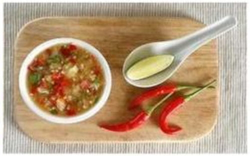
Sos chili

23

Owoce morza

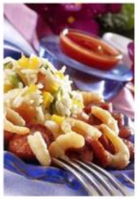
Ryż z krewetkami

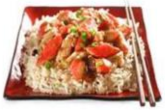
Paluszki krabowe

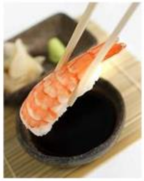
Sushi

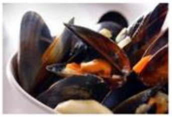
Mule

24
