# Supplementary figures and images for: Individual characteristics, including olfactory efficiency, age, body mass index, smoking and the sex hormones status, and food preferences of women in Poland
Source: PeerJ. 2022 Jun 15;10:e13538. doi: 10.7717/peerj.13538 (PMC9206430; doi:10.7717/peerj.13538)

**Określ, w jakim stopniu przyjemny jest dla Ciebie ten posiłek?**

**1. Potrawy z ryb**

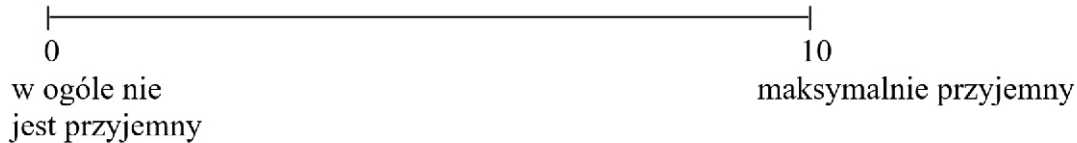

**2. Potrawy z jajek**

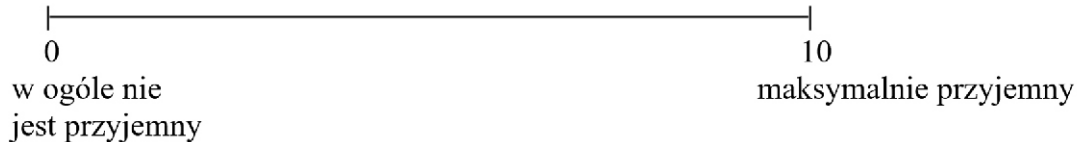

Supplement: Supplemental Information 2 [file peerj-10-13538-s002.pdf]

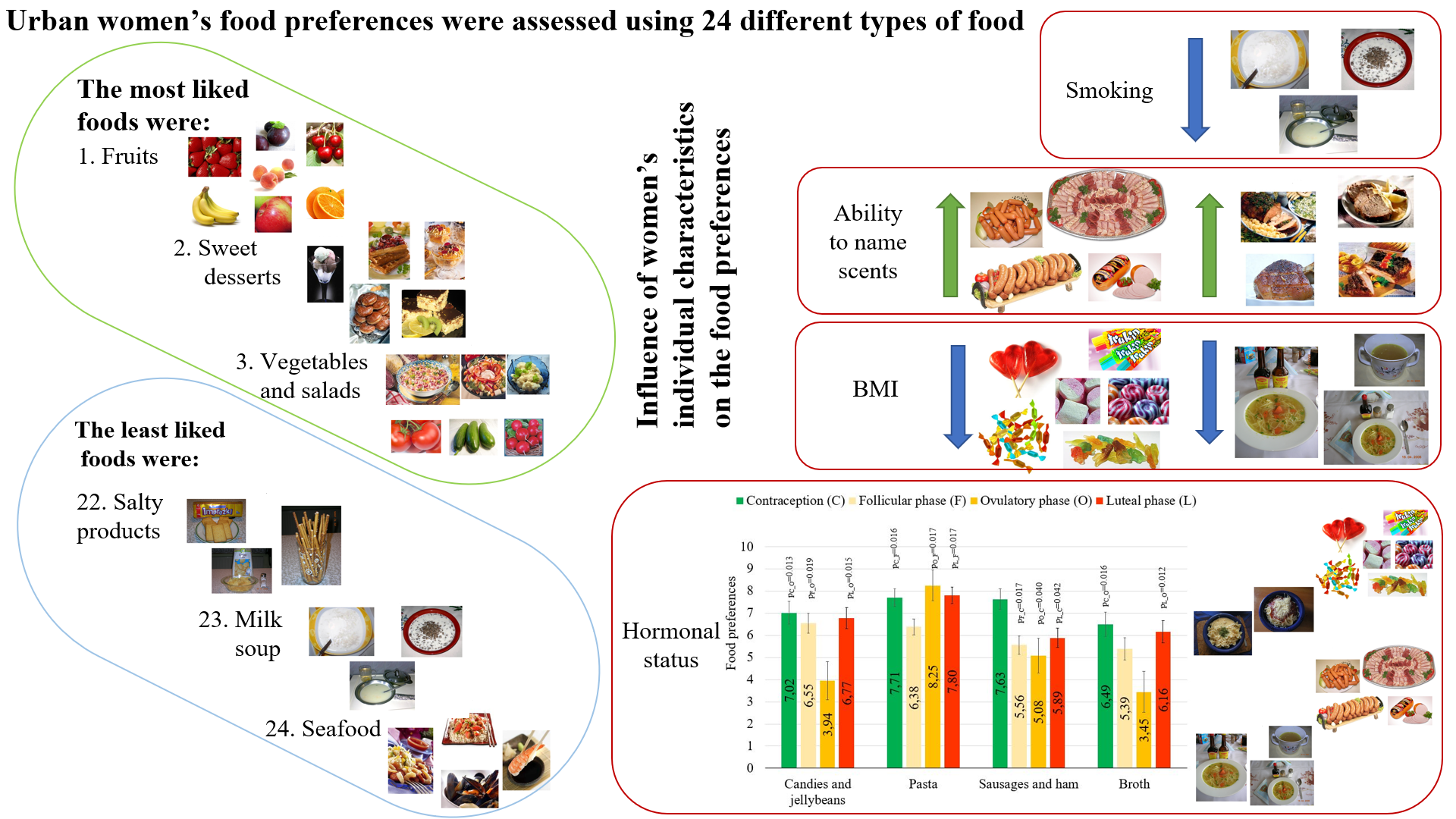

Supplement: Supplemental Information 4 [file peerj-10-13538-s004.png]
