# Supplementary material for: Individual characteristics, including olfactory efficiency, age, body mass index, smoking and the sex hormones status, and food preferences of women in Poland
Source: PeerJ. 2022 Jun 15;10:e13538. doi: 10.7717/peerj.13538 (PMC9206430; doi:10.7717/peerj.13538)
Supplement: Supplemental Information 3 [file peerj-10-13538-s003.docx]

**Suplement 3**. Regression models in which at least one predictor variable (age, BMI, pack-years, olfactory sensitivity (dilution step), ability to name scents, hormonal status: M- menopause C- contraception, F- follicular phase, O- ovulatory phase, L- luteal phase) affected the declared pleasure derived from eating a certain type of food. B - Unstandardized regression coefficients; PU - confidence interval; R^2^_c_ - multiple determination coefficient; eta^2^-effect size; t / F – t or F Statistic; p – level of significance.

| Dish | R^2^_sk_ | Predictor variables | B | PU | | t / F | eta^2^ | p |
| --- | --- | --- | --- | --- | --- | --- | --- | --- |
| Fruits | 0.07 | Age | .05 | < .01 | .10 | 1.99 | .02 | .048 |
|  |  | BMI | -.06 | -.13 | .02 | -1.53 | .01 | .127 |
|  |  | Pack-years | -.02 | -.08 | .03 | -.87 | < .01 | .384 |
|  |  | Olfactory sensitivity  (dilution step) | .06 | -.03 | .14 | 1.32 | .01 | .189 |
|  |  | Ability to name scents | -.07 | -.32 | .17 | -.60 | < .01 | .549 |
|  |  | Hormonal status (M/C/F/O/L) |  |  |  | .36 | < .01 | .549 |
| Bread | 0.11 | Age | .01 | -.05 | .07 | .30 | < .01 | .763 |
|  |  | BMI | -.09 | -.19 | < .01 | -2.01 | .02 | .046 |
|  |  | Pack-years | -.09 | -.16 | -.02 | -2.40 | .03 | .017 |
|  |  | Olfactory sensitivity  (dilution step) | .09 | -.02 | .20 | 1.61 | .02 | .108 |
|  |  | Ability to name scents | -.06 | -.37 | .26 | -.36 | < .01 | .717 |
|  |  | Hormonal status (M/C/F/O/L) |  |  |  | 2.57 | .06 | .040 |
| Fish dishes | 0.09 | Age | .03 | -.05 | .11 | .82 | <.01 | .412 |
|  |  | BMI | -.05 | -.17 | .07 | -.86 | <.01 | .389 |
|  |  | Pack-years | -.10 | -.19 | -.01 | -2.13 | .03 | .034 |
|  |  | Olfactory sensitivity  (dilution step) | .02 | -.12 | .15 | .25 | <.01 | .806 |
|  |  | Ability to name scents | .23 | -.16 | .63 | 1.16 | .01 | .246 |
|  |  | Hormonal status (M/C/F/O/L) |  |  |  | .900 | .021 | .465 |
| Candies and jellybeans | 0.08 | Age | < .01 | -.10 | .09 | -.06 | < .01 | .956 |
|  |  | BMI | -.14 | -.28 | < .01 | -2.04 | .02 | .043 |
|  |  | Pack-years | -.06 | -.17 | .04 | -1.21 | .01 | .230 |
|  |  | Olfactory sensitivity  (dilution step) | .03 | -.13 | .18 | .31 | < .01 | .756 |
|  |  | Ability to name scents | .15 | -.31 | .62 | .64 | < .01 | .523 |
|  |  | Hormonal status (M/C/F/O/L) |  |  |  | 1.59 | .04 | .179 |
| Broth | 0.09 | Age | .05 | -.05 | .14 | 1.01 | .01 | .315 |
|  |  | BMI | -.14 | -.28 | <.01 | -2.03 | .02 | .044 |
|  |  | Pack-years | .05 | -.05 | .16 | .98 | .01 | .326 |
|  |  | Olfactory sensitivity  (dilution step) | -.03 | -.20 | .13 | -.42 | <.01 | .671 |
|  |  | Ability to name scents | .08 | -.40 | .55 | .32 | <.01 | .748 |
|  |  | Hormonal status (M/C/F/O/L) |  |  |  | 2.36 | .05 | .055 |
| Sausages and ham | 0.12 | Age | .03 | -.05 | .11 | .80 | < .01 | .423 |
|  |  | BMI | < .01 | -.12 | .11 | -.05 | < .01 | .958 |
|  |  | Pack-years | -.08 | -.17 | .01 | -1.70 | .02 | .092 |
|  |  | Olfactory sensitivity  (dilution step) | < .01 | -.14 | .14 | < .01 | < .01 | .999 |
|  |  | Ability to name scents | .45 | .06 | .85 | 2.26 | .03 | .025 |
|  |  | Hormonal status (M/C/F/O/L) |  |  |  | 3.51 | .08 | .009 |
| Crisps | 0.13 | Age | -.15 | -.23 | -.06 | -3.35 | .06 | .001 |
|  |  | BMI | .03 | -.10 | .16 | .45 | < .01 | .652 |
|  |  | Pack-years | .01 | -.09 | .11 | .24 | < .01 | .812 |
|  |  | Olfactory sensitivity  (dilution step) | .05 | -.09 | .20 | .70 | < .01 | .485 |
|  |  | Ability to name scents | .01 | -.41 | .44 | .06 | < .01 | .953 |
|  |  | Hormonal status (M/C/F/O/L) |  |  |  | 1.24 | .03 | .296 |
| Milk soups | 0.04 | Age | .10 | .01 | .20 | 2.19 | .03 | .030 |
|  |  | BMI | -.06 | -.19 | .08 | -.80 | < .01 | .422 |
|  |  | Pack-years | .02 | -.08 | .13 | .43 | < .01 | .669 |
|  |  | Olfactory sensitivity  (dilution step) | .03 | -.13 | .19 | .37 | < .01 | .713 |
|  |  | Ability to name scents | .07 | -.39 | .53 | .30 | < .01 | .766 |
|  |  | Hormonal status (M/C/F/O/L) |  |  |  | 1.12 | .03 | .348 |
| Milk drinks | 0.09 | Age | .03 | -.05 | .12 | .80 | < .01 | .423 |
|  |  | BMI | -.05 | -.18 | .07 | -.85 | < .01 | .395 |
|  |  | Pack-years | .03 | -.06 | .13 | .65 | < .01 | .514 |
|  |  | Olfactory sensitivity  (dilution step) | .17 | .03 | .31 | 2.35 | .03 | .020 |
|  |  | Ability to name scents | .01 | -.40 | .43 | .07 | < .01 | .944 |
|  |  | Hormonal status (M/C/F/O/L) |  |  |  | 1.44 | .03 | .222 |
